# A potential regulatory network underlying distinct fate commitment of myogenic and adipogenic cells in skeletal muscle

**DOI:** 10.1038/srep44133

**Published:** 2017-03-09

**Authors:** Wenjuan Sun, Ting He, Chunfu Qin, Kai Qiu, Xin Zhang, Yanhong Luo, Defa Li, Jingdong Yin

**Affiliations:** 1 State Key Lab of Animal Nutrition, College of Animal Science and Technology, China Agricultural University, Beijing, 100193, China

## Abstract

Mechanism controlling myo-adipogenic balance in skeletal muscle is of great significance for human skeletal muscle dysfunction and myopathies as well as livestock meat quality. In the present study, two cell subpopulations with particular potency of adipogenic or myogenic differentiation were isolated from neonatal porcine longissimus dorsi using the preplate method to detect mechanisms underlying distinct fate commitment of myogenic and adipogenic cells in skeletal muscle. Both cells share a common surface expression profile of CD29^+^CD31^−^CD34^−^CD90^+^CD105^+^, verifying their mesenchymal origin. A total of 448 differentially expressed genes (DEGs) (FDR < 0.05 and |log_2_ FC| ≥ 1) between two distinct cells were identified via RNA-seq, including 358 up-regulated and 90 down-regulated genes in myogenic cells compared with adipogenic cells. The results of functional annotation and enrichment showed that 42 DEGs were implicated in cell differentiation, among them PDGFRα, ITGA3, ITGB6, MLCK and MLC acted as hubs between environment information processing and cellular process, indicating that the interaction of the two categories exerts an important role in distinct fate commitment of myogenic and adipogenic cells. Particularly, we are first to show that up-regulation of intracellular Ca^2+^-MLCK and Rho-DMPK, and subsequently elevated MLC, may contribute to the distinct commitment of myogenic and adipogenic lineages via mediating cytoskeleton dynamics.

The total fat content within skeletal muscle has been closely associated with metabolic disorders in humans^1^ as well as meat quality in farm animal production^2^. Fat deposition in muscle can be in the form of intramyocellular lipid droplets within muscle fibres, and lipid stored in adipocytes interspersed in the perimysial space or within fascicles^3^, and the latter contributes the major part to the total fat content in skeletal muscle^4^,^5^.

Myocytes and adipocytes including intramuscular adipocytes, originated from a common mesenchymal stem cells (MSCs) that has potential to differentiate into several distinct lineages^6^,^7^,^8^,^9^. Myogenesis and adipogenesis in skeletal muscle occur competitively in the same microenvironment^6^,^10^. The appearance of adipocytes in skeletal muscle was supposed to be associated with default of the expression of transcription factors that direct myogenic lineage commitment, which led to a phenotypic switch into the adipogenic lineage^11^. Thus, it is of great significance to clarify the regulatory network that controls distinct fate commitment of myogenic and adipogenic cells, which influences the origin and number of intramuscular adipocytes.

The commitment of stem cells to a particularly lineage is highly context dependent on the interactions of multiple extracellular signals^12^. Several factors, including cytokines, adhesion molecules, integrins, and transcriptional regulators, have been identified to be involved in the mediation of MSCs commitment to a particular lineage^12^,^13^,^14^,^15^. It has been reported that RhoA plays a key role in MSCs commitment into either adipocytes or myocytes regulated by these factors^12^. Furthermore, Rho superfamily GTPases (Rac1 or RhoA) have also been implicated in switching MSCs commitment to the chondrogenic versus myogenic or adipogenic versus osteogenic lineage through mediating cytoskeleton change^16^,^17^. Knowledge of mechanisms of skeletal muscle-derived mesenchymal cell commitment into the myogenic or adipogenic lineage is crucial for understanding skeletal muscle development and intramuscular fat deposition. However, it remains unclear.

In this study, adipogenic and myogenic cells were isolated from neonatal porcine skeletal muscle by the preplate method, and their differentiation potential, lineage origin and RNA expression profile were characterized. Based on functional annotation and enrichment analysis of DEGs, and the elevated intracellular Ca^2+^ concentration in myogenic cells, we are first to identified that different mediation of Rho-DMPK and Ca^2+^-MLCK by extracellular signal molecules PDGFs and ECMs, and subsequently MLC expression, might contributed to distinct fate commitment to myogenic or adipogenic lineage via remodeling the cytoskeleton dynamics.

## Results

### Isolation of myogenic and adipogenic cells from neonatal porcine skeletal muscle

**Figure 1:**
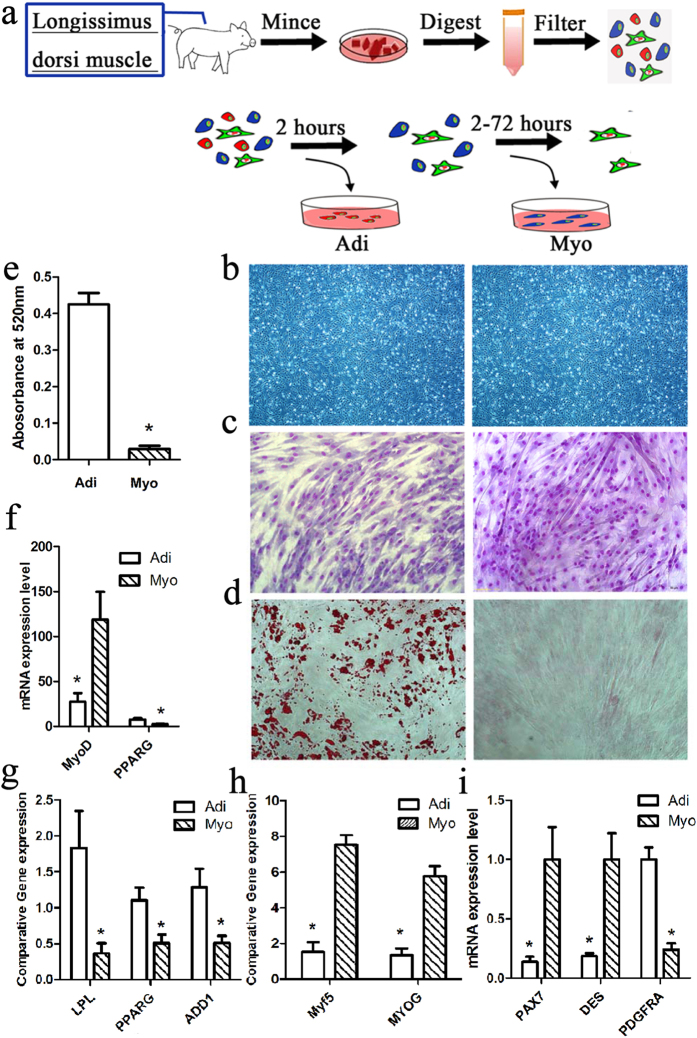
Isolation and identification of adipogenic (Adi) and myogenic (Myo) cells from porcine skeletal muscle by preplate technique. (**a**) Procedure of isolating adipogenic (Adi) and myogenic (Myo) cells. (**b**) Morphology of adipogenic (Adi) and myogenic (Myo) cells at passage 3, the magnification of microscope is (4 * 10). (**c**) porcine skeletal muscle-derived adipogenic (Adi) and myogenic (Myo) cells were grown on collagen-I coated dishes in in myogenesis induction medium for 5 days, cell were fixed, stained with Gimsa, the magnification of microscope is (10 * 10) (**d**) or in adipogenesis induction medium for 10 days, cell were fixed, stained with oil-red O, the magnification of microscope is (10 * 10). (**e**) Quantitative analysis of lipid droplet by oil-red O OD. Measurement of adipogenic (Adi) and myogenic (Myo) cells that after adipogenesis induction at d10. Values are means (n = 3) ± SEM, *represent significant difference between adipogenic (Adi) and myogenic (Myo) cells with *p* < 0.05. (**f**) Gene expression (n = 3) analysis of MyoD and PPARG in pre-induction adipogenic and myogenic cells by RNA-sequencing. (**g**) and (h) RT-qPCR analysis of adipogenesis- and myogenesis-related gene expression after adipogenic or myogenic induction. The data of each type cells comes from three replicates. (**i**) RT-qPCR analysis gene expression of myogenic markers (PAX7 and Desmin) and adipogenic marker (PDGFRα) and in pre-induction adipogenic (Adi) and myogenic (Myo) cells. The data of each type cells comes from three replicates. The procedure of isolating adipogenic (Adi) and myogenic (Myo) cells draw by Adobe Photoshop (CS5).

**Figure 2:**
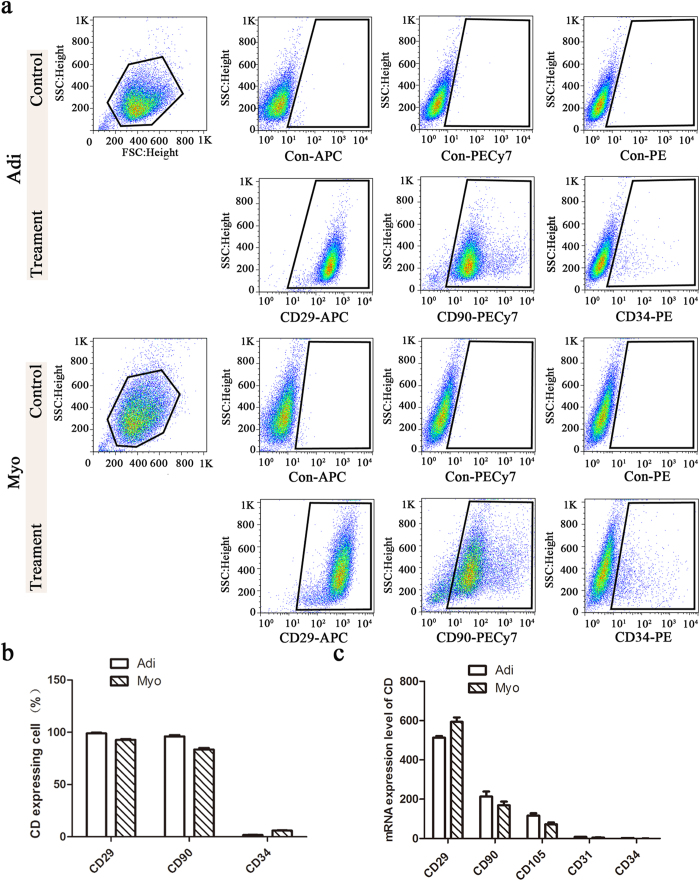
Expression of mesenchymal surface marker in porcine skeletal muscle-derived adipogenic and myogenic cells. (**a**) Representative flow cytometry analyses of adipogenic adipogenic (Adi) and myogenic (Myo) cells. (**b**) Percentages of positive cells for CD29 (APC), CD90 (PE-Cy7), and CD34 phycoerythrin (PE) were determined by flow cytometry. (**c**) Gene expression level (n = 3) of CD29, CD90, CD105, CD34, and CD31 analyzed by RNA-seq. The data of each type cells comes from three replicates. FlowJo (Tree Star) was used for all flow cytometry analysis.

Skeletal muscle-derived adipogenic (adherence to collagen I-coated dishes within 2 hours) and myogenic cells (adherence to collagen I-coated dishes during 2–74 hours) were isolated using the preplate method based on their different adherent capacity to collagen I-coated dishes (Fig. 1a). Pre-induction cells were identified in bright field of microscopy by their typical spindle shape (Fig. 1b). Upon myogenic induction, myogenic cells committed to multi-nuclei myotubes and myogenic-specific genes such as myoblast determination protein 1 (MyoD1) and myogenic factor 5 (Myf5) were highly expressed. However, no myogenic activity was seen in adipogenic cells (Fig. 1c,h). On the other hand, when treated with adipogenic induction, adipogenic cells differentiated into mature adipocytes, adopting a round shape (Fig. 1d), accumulating lipid (as shown by Oil-red O stained) (Fig. 1e), and expressing high levels of mRNA abundance of adipocyte-specific genes, such as lipoprotein lipase (LPL), peroxisome proliferator-activated receptor γ (PPARγ) and sterol regulatory element binding transcription factor 1 (ADD1 or SREBP-1) (Fig. 1g). While, little or no adipogenic activity was evident in myogenic cells (Fig. 1d,e,g). In agreement with the above results, RNA-seq data showed that adipogenic cells had a higher level of mRNA expression of PPARγ, a key transcription factor for adipogenesis, compared with myogenic cells (FDR < 0.05), and a lesser level of MyoD1, a key transcription factor for myogenesis (FDR < 0.05) (Fig. 2f). Additionally, RT-qPCR data showed that myogenic cells possessed higher mRNA expression level of myogenic markers genes, with 10.76 fold-change of PAX7 and 5.56 fold-change of desmin compared with adipogenic cells. Adipogenic cells had a very higher mRNA expression level of adipogenic marker PDGFRα with 4.57 fold change (adipogenic vs myogenic cells) (Fig. 1i and Table S1). Importantly, the results of cell differentiation induction indicated that adipogenic cells committed to an adipogenic lineage cannot convert into myogenic lineage, while myogenic cells committed to a myogenic lineage cannot convert into adipogenic lineage. That is, we isolated two cells with distinct differentiation potential from porcine skeletal muscle.

### Mesenchymal origin of adipogenic and myogenic cells

Both myogenic and adipogenic cells were positive for MSCs markers CD29, CD90 and negative for hematopoietic lineage markers CD34 (Fig. 2a). There was no difference between myogenic and adipogenic cells in the expression level of above cell surface markers (Fig. 2b). Consistent with these flow cytometry data, RNA-seq data showed that both myogenic and adipogenic cells had high expression levels of MSCs markers CD29, CD90 and CD105 and very low expression levels of hematopoietic lineage marker CD34 and endothelial lineage marker CD31 (Fig. 2c). These results indicated that mesenchymal origin of myogenic and adipogenic cells, but not hematopoietic or endothelial lineage.

### Global gene expression profile and differentially expressed genes of myogenic and adipogenic cells

**Figure 3:**
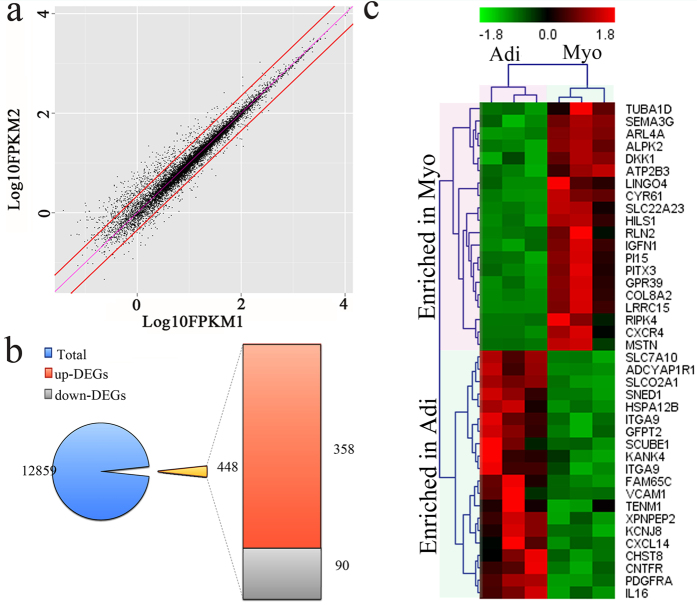
DEGs between adipogenic and myogenic cells based on the RNA-sequencing data. (**a**) Genes were categorized into DEGs (plots that off the two red line) based on their position to the diagonal (FPKM1 represent adipogenic (Adi) cells, FPKM2 represent myogenic (Myo)). (**b**) Overview of DEGs numbers between adipogenic (Adi) and myogenic (Myo) cells. (**c**) Hierarchical clustering of top 40 DEGs (top 20 genes with either higher or lower expression in myogenic compared to adipogenic cells) between adipogenic (Adi) and myogenic (Myo) cells. The data of each type cells comes from three replicates. Heat-maps were generated by MeV (4.9.0).

After filtration of raw data, 75.76 M clean reads were obtained, with the number of sequence reads range from 11.09 M to 14.06 M single paired reads for all 6 samples, and Q30 was above 95.24% (Supplementary Table S2). Above 84.01% clean reads of each sample were mapped to Sus scrofa genome (Suscrofa10.2). By comparing mapped reads against the ensemble genome annotation, a total of 12859 transcripts in adipogenic and myogenic cells in all 6 samples were detected. A total of 448 genes showed differential expression (FDR < 0.05 and |log_2_ FC| ≥ 1), of which 358 genes were up-regulated and 90 genes were down-regulated in the myogenic cells (Fig. 3a,b and Supplementary Table S3).

The top 20 DEGs with the highest or lowest fold-change identified by RNA-seq were shown in Fig. 3c. Intriguingly, among genes enriched in myogenic cells, growth/differentiation factor 8 (GDF8) or myostatin (MSTN), which plays an important role with other co-regulatory factors in muscle development^18^, was with the highest fold-change (36.49-fold). While among genes enriched in adipogenic cells, family with sequence similarity 65, member C (FAM65C) was the gene with the highest fold change (6.81-fold). Protein FAM65C, a member of extracellular region part, regulates cellular process in response to stimulus. Its function is obscured yet, whereas its family member FAM65B has been implicated in myogenic cell differentiation^19^.

**Figure 4:**
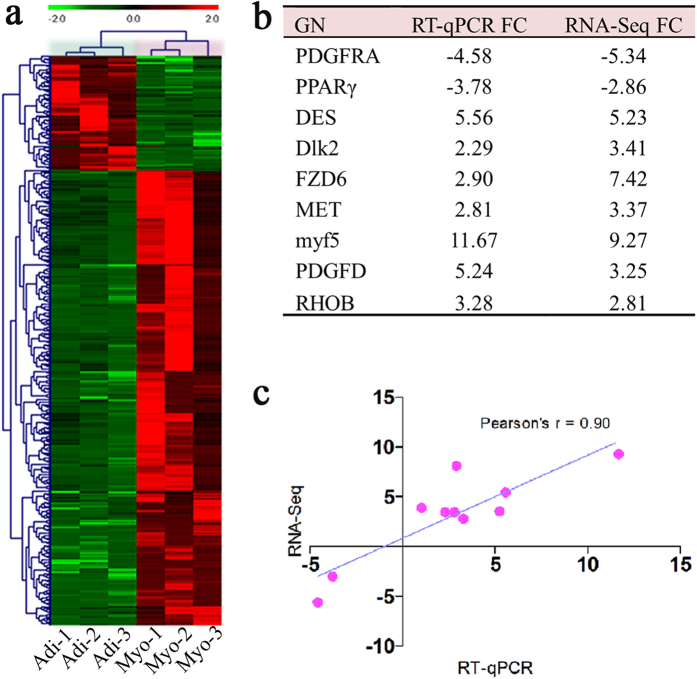
RNA-seq examination via hierachization clustering and validation via RT-qPCR. (**a**) All DEGs between adipogenic (Adi) and myogenic (Myo) cells based hierachization clustering. (**b**) Fold change (FC) of 9 selected DEGs calculated with mRNA expressions of myogenic vs adipogenic cells determined via RNA-seq (n = 3) and RT-qPCR (n = 3). (**c**) The correlation of fold changes in gene expression between the RNA-seq and RT-qPCR (Pearson correlation analysis were conducted by SAS 9.1). Heat-maps were generated by MeV (4.9.0).

To evaluate the mRNA expression consistency of the same type cells among different biological replicates and the variance between adipogenic and myogenic cells, hierachization clustering was carried out for all DEGs. DEGs-based hierarchical clustering demonstrated that adipogenic or myogenic cells from the three individual pigs belonged to the same cluster, and results from transcriptome profiles of biological replicates were very consistent with distinct gene expression profiles between adipogenic and myogenic cells (Fig. 4a), which indicates the validity of results of the gene expression analysis.

### Validation of DEGs using RT-qPCR

To validate the expression profiles of the DEGs identified from the high-throughput RNA-seq, nine genes were randomly selected for RT-qPCR assays. Fold change of selected DEGs were calculated with mRNA expressions level of myogenic vs adipogenic cells detected via RNA-seq and RT-qPCR (Fig. 4b). Relative gene expression patterns of the selected genes were significantly correlated between the RT-qPCR and RNA-seq (Pearson’s r = 0.90) (Fig. 4c). Therefore, the RT-qPCR result largely demonstrated the reliability of RNA-seq data.

### GO and KEGG enrichment analysis of DEGs

Gene ontology (GO) and Kyoto Encyclopedia of Genes and Genomes (KEGG) enrichment analysis with the 448 DEGs were performed. In GO analysis, DEGs were classified into functional categories, including 156 biological process terms, 46 cellular component terms and 41 molecular function terms under the filtering option with *q* < 0.05 (Supplementary Table S4). Enriched terms that closely associated with interaction between cells differentiation and micro-environment which influences cell fate commitment, can be divided into five categories (Table 1), including: (1) cell differentiation and fate determination (2) the alteration of cell component (including extracellular matrix, focal adhesion, cytoskeleton) (3) binding and cell adhesion (4) signaling and signaling transduction (5) proteins modification (polymerization and phosphorylation).

**Figure 5:**
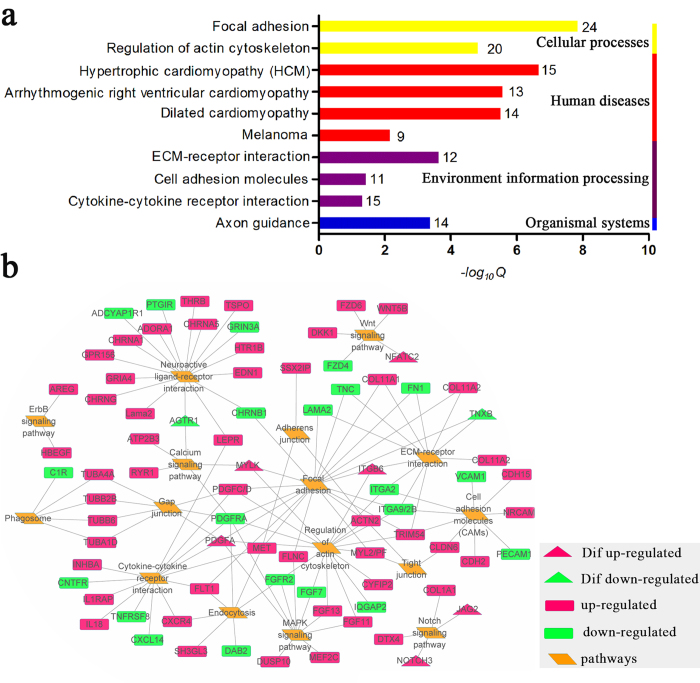
KEGG analysis of DEGs and their relationship with cell differentiation. (**a**) Significantly enrichment pathways (*q* < 0.05) based on identified DEGs. (The number in the right of columns represented the number of DEGs that involved in those enrichment pathway). (**b**) Transcriptional regulatory network of DEGs in key KEGG pathways, which included by environmental information processing and cellular process. Diamonds represent KEGG pathways, rectangles represent genes involved in the KEGG pathways and the triangle represent DEGs related to cell differentiation. The colour of the nodes represents gene expression status (red means genes up-regulated in Myo, green means down-regulated in Myo). Networks were visualized by Cytoscape (v3.2.1).

In KEGG pathway analysis, we were able to assign 109 unique KEGG orthologs to 144 DEGs (24.3%) (Supplementary Table S5). The top 50 pathways were mainly clustered into 5 categories: cellular process, environment information processing, human disease, organismal systems and metabolism (Supplementary Figure S1). We identified 10 significantly enriched KEGG pathways (*q *<* *0.05) (Fig. 5a). Human cardiac muscle disease related pathways such as cardiomyopathies, including hypertrophic cardiomyopathy (HCM), arrhythmogenic right ventricular cardiomyopathy (ARVC) and dilated cardiomyopathy (DCM), are well known for their connection to Extracellular matrix (ECM) -receptor interaction pathways, and most DEGs involved in HCM, ARVC, and DCM are ECM-receptor interaction-related genes. Aside from human cardiac muscle disease related pathways, the top three significantly enriched pathways were focal adhesion, ECM-receptor interaction, and regulation of actin cytoskeleton. Both focal adhesion and regulation of actin cytoskeleton belong to the cellular process category. ECM-receptor interaction is included in environment information processing (Fig. 5a). Furthermore, 47 DEGs, enriched in the alteration of cell component category that includes extracellular matrix, focal adhesion, cytoskeleton (Supplementary Table S4), is the molecular basis for regulation of cytoskeleton pathway.

### Interaction between environment information processing and cellular process in fate commitment of myogenic and adipogenic cells

A total of 81 DEGs were enriched in the categories of the environment information processing and cellular process. Most of these DEGs (57 out of 81) were up-regulated while 24 DEGs were down-regulated in myogenic cells compared with adipogenic cells (Supplementary Table S6). Among them, eight DEGs were assigned to “cell differentiation” of GO, including type-1 angiotensin II receptor (AGTR1), tenascin-X precursor (TNXB), Myosin light chain kinase (MLCK), platelet-derived growth factor subunit A (PDGFA), integrin beta 6 (ITGB6), protein jagged-2 (JAG2), neurogenic locus notch homolog protein 3 (NOTCH3), nuclear factor of activated T-cells, cytoplasmic 2 (NFATC2) (Table 1). For fully understanding their interaction in distinct fate commitment of myogenic and adipogenic cells in skeletal muscle, cytoscape (v3.2.1) was applied to depict a putative molecular network among the DEGs involved as well as the total of 16 signaling pathways, of which nine pathways involved in environment information processing and seven pathways contained in cellular process (Fig. 5b). It is interesting that PDGFA, PDGFRα, MLCK, ITGB6, ITGA9 and ITGA2B, acted as hubs among the signaling pathways, including focal adhesion, ECM-receptor interaction, regulation of actin cytoskeleton, cytokine-cytokine receptor interaction, calcium signaling pathway, as well as MAPK signaling pathway (Fig. 5b).

Focal adhesion, as a transmembrane bridge, links ligands (ECMs and cytokines) and the cytoskeleton. In present study, significant numbers of identified DEGs were implicated in this process (Supplementary Table S6), including (1) growth factors and related receptors, such as fibroblast growth factors (FGF7, FGF9, FGF11, FGF13) and fibroblast growth factor receptor (FGFR2), platelet-derived growth factors (PDGFA, PDGFC, PDGFD) and platelet-derived growth factor receptor (PDGFRα), and growth factors receptors (FLT1, MET); (2) components of cytoskeleton, ECMs and focal adhesion, such as myosin light chains (MLC, including MYLPF and MYL2), myosin light chain kinase (MLCK), integrins (ITGA2, ITGA2B, ITGB6, ITGA9, TRIM54), tenascins (TNXB, TNC, FN1), collagens (COL11A1, COL11A2), laminin (LAMA2), actinin (ACTN2) and filamin (FLNC); (3) Ras superfamily GTPase (RHOB), Rho guanine nucleotide exchange factors (RhoGEF, Rho family activator) and ras GTPase-activating-like protein (DLC, Rho family inhibitor).

**Figure 6:**
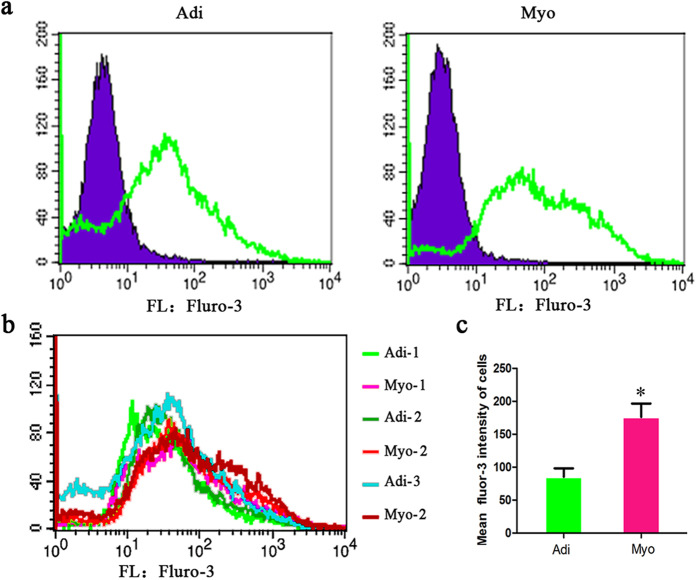
Intracellular calcium concentration differed in adipogenic and myogenic cells of porcine. (**a**) Average Ca^2+^ signals of adipogenic (Adi) and myogenic cells (Myo) compared with unlabled control (the blue represented unlabled control, the green represented fluro-3 labled cell). (**b**) Average Ca^2+^ signals of adipogenic (Adi) and myogenic cells (Myo) from porcine skeletal muscle, the data of every group comes from three replicates. (**c**) Mean fluro-3 intensity of adipogenic (Adi) and myogenic cells (Myo) from porcine skeletal muscle, *significant difference between adipogenic (Adi) and myogenic cells (Myo) with *p* < 0.05.

Interestingly, quite a few DEGs were enriched into the calcium pathways or related functions. We discovered that five DEGs were involved in the calcium signaling pathway (Supplementary Table S6), of which sarcoplasmic reticulum calcium (Ca^2+^) release channel (RYR1), plasma membrane calcium ATPase (ATP2B3 or PMCA), and MLCK were up-regulated in myogenic cells compared with adipogenic cells, while cytokine receptor (PDGFRα) and G-protein coupled receptor (AGTR1) were significantly down-regulated. In addition, other six DEGs were assigned to the “calcium-mediated signaling” (GO: 0019722, q = 0.006) and a total of 32 DEGs were assigned to the “calcium ion binding” (GO: 0005509, q = 0.008) (Supplementary Table S7). Furthermore, the elevation of intracellular Ca^2+^ was also observed in myogenic cells compared with adipogenic cells (Fig. 6). Elevated Ca^2+^ concentrations detected in myogenic cells may be associated with the altered gene expression in the following potential ways: Cav1-RYR1 mediated, PDGF-PDGFR-PLCγ or GPCR- PLCβ-IP3R mediated Ca^2+^ release from intracellular compartment, and ATP2B3 mediated the exchange of Ca^2+^ with extracellular environment (Supplementary Figure S2).

Significantly, several key DEGs were simultaneously involved in multiple key signaling pathways that belong to the categories of environment information processing and cellular process. For instance, PDGFRα, down-regulated in myogenic cells relative to adipogenic cells, is not only involved in the regulation of actin–myosin pathway (Supplementary Figures S3 and S4) based cytoskeleton dynamics, but also acts as an inducer of PLCγ in the calcium signaling pathway (Supplementary Figure S2). MLC, up-regulated in myogenic cells in the present study, plays a vital role in regulation of the actin cytoskeleton pathway after being activated by dystrophia myotonica protein kinase (DMPK) or MLCK through phosphorylation (Supplementary Figures S3 and S4), simultaneously, it is also involved in the calcium signaling pathway after being activated by MLCK (Supplementary Figure S2). These results are another demonstration that PDGFRα, MLCK and MLC acted as hubs in connecting genes involved in the environment information processing (ECM-receptor interaction and calcium signaling pathway) and cellular process (regulation of actin cytoskeleton) related pathways.

## Discussion

Myocytes and adipocytes are derived from a common origin MSCs. However, mechanisms that regulate adipogenic or myogenic lineage commitment remain unclear. The present study is the first to show the panoramic distinction of gene expression profile based on RNA-seq between adipogenic and myogenic cells, which would help to understand the potential regulatory network that underpins the distinct fate commitment of myogenic versus adipogenic lineage in skeletal muscle.

Magnetic cell sorting and fluorescent activated cell sorting (FACS) have been developed to sort subpopulations of stem cells on the basis of the cell surface marker profiles with some degree of success^20^,^21^,^22^,^23^,^24^. It has been shown that PDGFRα, Sca1, CD34 or CD15 are typically used as specific cell surface markers for adipogenic cells^21^,^22^, while CD56 is extensively used as a marker for identification and isolation of myogenic cells from human skeletal muscle, although CD56^+^ cell subpopulation was reported to exhibit both myogenic and adipogenic potential^23^. In pigs, cell surface marker-dependent isolation methods, have not been established yet due to limited knowledge on cell surface markers of muscle-derived cells and few available commercialized antibodies for pigs. Preplate technique, a surface marker-independent method, has been developed to isolate skeletal muscle-derived cell fraction in various species based on cell adhesion characteristics to collagen-coated tissue culture dishes^25^,^26^,^27^. It has been well documented that rapidly adhering cell fraction (within 2 h of seeding) derived from human skeletal muscle is mainly composed of fibroblasts, with the capability of adipogenic differentiation^28^, whereas a slowly adhering cell fraction (during 2–72 hours of seeding) is myogenic cells^25^, including activated and quiescent satellite cells, which were Pax7, Desmin, MyoD, Myf5 postive^25^,^28^,^29^. In this study, we applied preplate technique to obtained adipogenic and myogenic cells that have distinct differentiation fate and cannot convert their commitment into opposing lineages.

In human skeletal muscle, adipogenic-myogenic bipotential cells (CD34^+^CD15^+^CD56^+^), adipogenic cells (CD34^+^CD15^+^CD56^-^) and myogenic cells (CD34^+^CD15^−^CD56^+^) were positive for typical MSCs or progenitor cell surface markers of CD44, CD146, CD49, CD90, negative for the lineage markers of CD45, CD106, CD117, CD133, and STRO-1, and expressed a low level of the endothelial marker CD31^21^. It was also reported that hematopoietic stem cells (HSC) demonstrate some level of myogenic potential within the bone marrow and other hematopoietic tissues^30^. Accordingly, in this study, RNA-seq results indicated that both myogenic and adipogenic cells expressed CD29, CD90, CD105 (MSCs surface markers), but not CD34 (hematopoietic lineage marker) or CD31. This was also evident in flow cytometry analysis. On the basis of the cell surface marker expression results, we were able to demonstrate that adipogenic and myogenic cells isolated by the preplate technique from porcine skeletal muscle were derived from MSCs rather than hematopoietic stem cells or endothelial cells. However, mechanisms of how extracellular factors induce cytoskeleton dynamics that regulate distinct fate commitment of myogenic or adipogenic cells remain unclear.

It has been well established that cytoskeletal dynamics, based on actin-myosin complex that consists of actin filaments and myosin II heavy chains (MYHs), exerts profound roles in cell differentiation^16^,^17^. In the present study, a total of 30 DEGs encode proteins that are involved in ECM-receptor interaction, focal adhesion and regulation of cytoskeleton organization (Supplementary Figures S3, S4 and S5). Based on the functional annotation and enrichment analysis of DEGs between adipogenic and myogenic cells, we identified that the cellular process (regulation of cytoskeleton and focal adhension) and environment information processing (ECM-receptor interaction, cell adhesion molecules, neuroactive ligand-receptor interaction, cytokine-cytokine receptor interaction, and calcium signaling pathway in particular) were closely involved in the mediation of distinct fate commitment of adipogenic and myogenic lineage, which showed that cytoskeleton dynamics differentially expressed between myogenic and adipogenic cells.

Actin filaments are often anchored on integrins through focal-adhesion^31^. Focal adhesion is involved in the regulation of cytoskeleton through linking extracellular regions and the actin-myosin complex and receiving the extracellular signals from ligands (Cytokine and ECMs). A number of DEGs, such as collagens, laminins, tenascins, and their receptors integrins, were enriched in ECM-receptor interaction pathway (Supplementary Figure S5). Changes in the ECMs may influence the clustering of integrin and other cell adhesion molecules, thus altering the number and distribution of growth receptors for tyrosine kinase in focal adhesions^32^. Consistently, for instance, hepatocyte growth factor receptor (MET) was up-regulated while PDGFRα was down-regulated in myogenic cells compared with adipogenic cells, which was shown by previous study in which ECM-receptor interaction was involved in the depot-specific adipogenesis^33^. Therefore, differential regulation of cytoskeleton dynamics by ECMs and PDGFs exists in myogenic and adipogenic cells, through binding with their receptors PDGFR and integrins, respectively.

Rho GTPase protein family, including Rho, CDC42 and Rac, plays vital roles in actin-myosin complex organization through dystrophia myotonica protein kinase family comprising DMPK, ROCK, and MRCK^34^. Additionally, cytosolic DMPK participates in remodeling of the actomyosin cytoskeleton by increased phosphorylation status of MLC2 in proliferating and differentiating myoblasts^35^.

In the present study, we detected the up-regulation of the RhoGEF, RhoB and DMPK in myogenic cells compared with adipogenic cells. Therefore, we deduced that Rho-mediated cytoskeleton organization was differentially regulated in the two cells. Meanwhile, the extracellular signal molecules PDGFs and ECMs increased RhoB and subsequent DMPK expressions via two independent pathways, and thereby increase MLC expression (Supplementary Figure S3), which may partly contribute to distinct fate commitment of skeletal muscle-derived myogenic and adipogenic cells.

Myosin light chain (MLC) is activated by phosphorylation through kinase, such as DMPK or MLCK, and subsequently triggered alterations of cytoskeleton organize based on the actin-myosin^36^. On the other hand, elevated intracellular Ca^2+^ can directly or indirectly activate the MLCK activity, and subsequently activate MLC through phosphorylation (Supplementary Figure S2). Interestingly, in the present study, the interaction between the pathways environment information processing and cellular process was differentially mediated and the key elements including PDGFRα, RHO, and DMPK were up-regulated in myogenic cells (Supplementary Figures S2, S3 and S4). Furthermore, a few DEGs encoding the proteins including CaV1, RYR1 and IP3R that mediate intracellular Ca^2+^ concentration^37^ and key Ca^2+^-mediated pathways enriched were differentially expressed between myogenic and adipogenic cells. RYR1 mediated Ca^2+^ signaling has been shown to play an important role in muscle organ development and myogenesis^38^. Particularly, the present study is the first to provide the direct evidence that intracellular Ca^2+^ concentration elevated in myogenic cells compared with adipogenic cells.

**Figure 7:**
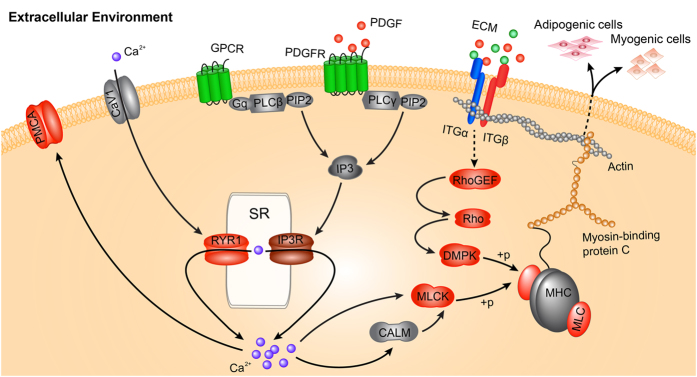
The potential regulatory network of fate determination of myogenic and adipogenic cells derived from porcine skeletal muscle. The colour of the nodes represent gene expression status (red means up-regulated in myogenic cells (Myo), green means down-regulated in Myo, blue means genes including both up- or down-regulated genes in Myo, IP3R was up-regulated in myogenic cells with FC = 1.36, FDR < 0.05). This model was visualized by Adobe Illustrator (CS5).

In addition, intracellular Ca^2+^ can also mediate stem cell differentiation into other lineages through activation of MAPK signaling pathway^39^,^40^. Consistently, MAPK pathway was enriched as differentially expressed between two cells (Supplementary Table S6, Supplementary Figure S1) and elevated intracellular Ca^2+^ might activate MAPK signaling pathway (Supplementary Figure S2). Therefore, it implied that the intracellular Ca^2+^ may play a vital role in distinct fate commitment of skeletal muscle-derived adipogenic and myogenic cells in porcine through the intracellular Ca^2+^-mediated MLCK-MLC-myosin-actin cytoskeleton signaling pathway (Fig. 7) or MAPK pathway.

## Conclusion

In our study, porcine skeletal muscle-derived myogenic and adipogenic cells were isolated and subjected to RNA-seq analysis to reveal the mechanisms underlying myogenic- and adipogenic cell commitment. A total of 448 DEGs were identified between myogenic and adipogenic cells. The most enriched pathways that are implicated with the distinct differentiation capacity of the cells involve environment information processing and cellular process. Remarkably, myogenic cells occupied the high expressed Rho-DMPK and intracellular Ca^2+^-MLCK pathways compared with adipogenic cells, which can remodel cytoskeleton organization through up-regulated the expression of MLC. It may partly contribute to the balance of adipogenesis versus myogenesis commitment in skeletal muscle. This study provided important insight into the distinct fate commitment of myogenic and adipogenic cells in skeletal muscle. However, further exploration is needed to elucidate the more exact molecular mechanisms.

## Methods

### Ethics statement

All procedures performed in this experiment were approved by China Agriculture University Animal ethics committee. All methods were performed in accordance with the relevant guidelines and regulations.

### Animals and tissue

Three 3-day-old newborn Yorkshire pigs (BW = 1.4 ± 0.2 kg) were acquired from the Beijing Pig Breeding Center and euthanized. Longissimus dorsi muscles were harvest from the last lib of pigs after slaughtered, and immediately rinsed in 75% ethanol for 3 s, then transferred to PBS (Hyclone, Thermo Scientific, Marietta, OH, USA), containing antibiotics (100 U/ml of penicillin and 100 mg/ml of streptomycin) (Gibco-BRL, Grand Island, NY, USA), and immediately transferred to a biological safety cabinet (HF-1100, HK, China) for cell isolation.

### Cell isolation and culturing

The preplate technique was conducted to obtain differential adhension capacity cells derived from porcine skeletal muscle. Using a previously described protocol^25^,^41^,^42^,^43^ with some modifications, single cell suspensions were obtained by enzymatic dissociation of around 1.5 g longissimus dorsi muscle from the last rib of euthanized pigs. Enzymatic dissociation was performed by serial digestion of approximately 3 mm^3^ muscle samples in 0.17% protease (Sigma-Aldrich, St. Louis, MO, USA) for 1 hour, 0.15% collagenase-type XI solution (Sigma-Aldrich Ltd) for 1 hour. The suspension was filtered through 100 μm and then 40 μm cell strainers (BD Biosciences, San Jose, CA, USA), pelleted by centrifugation, then resuspend in growth medium. The cell resuspension was plated in a 10 mm collagen-coated dish (Sigma-Aldrich Ltd) containing DMEM/F12 (Hyclone, Thermo Scientific, Marietta, OH), medium complemented with 10% FBS (Gibco-BRL Ltd), 2 mM glutamine (Gibco-BRL Ltd), antibiotics (100 U/ml of penicillin and 100 mg/ml of streptomycin), 5 ng/ml basic fibroblast growth factor (bFGF, PEPTECH, Burlington, MA, USA), and cultured at 37 °C under 5% CO_2_. After 2 hours, non-adherent cells contained in the supernatant were removed and transferred to a second plate for a period of 72 hours. Fresh medium was added to the first set of adherent cells which were labled as adipogenic cells. The second set of adherent cells during (2–74 h) were labled as myogenic cells.

### Adipogenic and myogenic cells differentiation and identification

When reaching 100% confluence, adipogenic differentiation was induced by adipogenic induction medium (DMEM supplemented with l μM dexamethasone, 0.5 mM 1-methyl-3-isobutylmethyl-xanthine, and 10 μg/ml insulin) (the reagents used in adipogenic induction medium were purchase from Sigma-Aldrich) for 3 days, then cells were cultured in adipogenic maintained medium (DMEM complemented with 10% FBS, 10 μg/ml insulin) for another 7 days. The detection of lipid droplets and assess the efficiency of adipogenic differentiation were conducted as previously described protocol^44^.

When cells reached 80–90% confluence, myogenic differentiation was induced by low serum medium that DMEM supplemented with 2% horse serum (Hyclone Ltd) for 5 days. Myotubes were identified as previously described protocol^45^.

### Flow cytometry

Flow cytometry was used to identify cell surface profiles of myogenic and adipogenic cells isolated from porcine skeletal muscle. Adherent cells were detached with 0.25% trypsin/0.01% EDTA (Sigma-Aldrich Ltd) for 40 s, and centrifuged at 1000 g for 5 min, then resuspend in PBS containing 2% FBS. Immunostaining of cells was carried out using manufacturers’ recommended antibody dilutions in a solution in PBS containing 2% FBS. Viable cells were then counted with an automated Cell Counter using the trypan-blue exclusion test (BIO RAD, CA, USA). Live cells (3 × 10^6^) were used for cell surface markers analysis. These cells were incubated with labeled monoclonal antibodies (mAb) CD29-APC (Clone MEM-101A, abcam, 1:100), CD90-PECy7 (Clone 5E10, bioscience, 1:100), and CD34-PE (Clone EP373Y, abcam, 1:100). All samples, protected from light, were incubated on ice for 30 min, washed in PBS 3 times, and fixed in 200 μl 1% paraformaldehyde^46^. After being fixed, labelled cells were analyzed with a flow cytometer (BD FACS Calibur, USA). Gated events (20,000) were collected for each sample. Debris and dead cells were excluded by forward scatter and side scatter. FlowJo (Tree Star) was used for all flow cytometry analysis. Data analysis were compared with negative control.

### cDNA library preparation and sequencing

Total cellular RNAs were extracted from adipogenic and myogenic cells (derived from the 3 pigs) using RNA extraction kit with DNA-elimator (Qiagen, Valencia, CA, Germany) under the manufacture instructions. RNA integrity and concentration were evaluated using a NanoDrop ND-1000 spectrophotometer (Thermo Scientific, Wilmington, DE, USA) and an Agilent 2100 Bioanalyzer (Agilent Technologies, Santa Clara, CA, USA). RNA samples were used in the study only when the A260/A280 ratio was >1.8, 28 S/18 S ratio >2.0. The cDNA library was constructed following the manufacturer’s instructions of NEB Next Ultra RNA Library Prep Kit (NEB, E7530) for Illumina and NEB Next Multiplex™Oligoes (NEB, E7500) for Illumina^47^. After quality control, six cDNA libraries constructed from adipogenic and myogenic cells, were sequenced on Illumina HiSeq™ 2500 sequencing platform.

### Global gene expression analysis and DEGs identification

Low quality reads, such as adaptor sequences, unknown nucleotides >5%, or low Q-value (≤20) base greater than 20%, were removed by perl script. The reads that met the levels of quality control were mapped to Sus scrofa genome (Sscrofa10.2) from Ensemble Sus (http://asia.ensembl.org/scrofa/Info/Index) using Tophat2 (2.0.12)^48^. Gene expression levels were estimated using FPKM values (fragments per kilo base of exon per million fragments mapped) by the Cufflinks software^49^. FDR < 0.05 and |log_2_ FC| ≥ 1 (FC represent fold change) were set as the threshold for DEG between adipogenic and myogenic cells^50^.

### RNA-seq examination via hierachization clustering and validation via RT-qPCR

Hierachization clustering analysis (conducted by MEV version 4.9.0) was used to visualize the variance of different individual samples for all DEGs. To validate the gene expression profiling result, quantitative real-time PCR was performed to analyze several selected genes expression. Total RNA was used synthesize cDNA using a Kit (CWbio, Beijing, China). With the synthesized cDNA of each sample, ABI 7500 Real-Time PCR system (Applied Biosystems, Foster City, CA, USA) and a quantitative real-time PCR kit (Takara, Japan) were used. The primers used in this experiment were listed in (Supplementary Table S8). GAPDH was used as an endogenous control. Relative gene expression level was calculated by 2−ΔΔCt method^51^. Student t-test was used to test hypotheses.

### Gene ontology and pathway analysis

Ensemble Gene IDs of identified DEGs were exploit for the following function enrichment analysis. Differentially expressed genes were annotated by comparison with protein databases (GO and KEGG^52^) via BLASTX. For GO analysis, Blast2 GO program were utilized mapped all of the annotated genes to GO terms in the database and counted the number of genes associated with each term^53^. KEGG pathways were assigned to the assembled sequences by perl script^54^. Cutoff criteria were set with Benjamini-Hochberg false discovery rate *q* < 0.05 for both GO and KEGG enrichment analysis.

### Intracellular calcium concentration measurement

Intracellular Ca^2+^ concentration was measured the by flow cytometry^55^ with some modification as follows. Adherent cells were detached and sampled, then incubated in 5 μg/mL fluo-3 acetoxymethyl ester (Cayman Chemical, Ann Arbor, MI, USA) for 45 min at 37 °C (protected from light). Cells were washed 3 times with PBS (containing 1% FBS) and centrifuged at 1000 rpm for 5 min. Then cells were resuspended in 200 μl PBS (containing 1% FBS). Calcium-bound fluo-3 has an emission maximum of 526 nm that was quantified after excitation with a 488-nm laser and collection through a 530/30 nm by flow cytometry and software (BD FACS Calibur, USA). Gated events (20,000) were collected for each sample. Debris and dead cells were excluded by forward scatter and side scatter. Mean fluorescence intensity was determined from the entire cell population.

### Statistics

Comparisons between adipogenic and myogenic cells were based on paired *t* test. Pearson analysis was used to correlate gene expression determined by RT-qPCR and RNA-seq.

## Additional Information

**How to cite this article**: Sun, W. *et al*. A potential regulatory network underlying distinct fate commitment of myogenic and adipogenic cells in skeletal muscle. *Sci. Rep.* **7**, 44133; doi: 10.1038/srep44133 (2017).

**Publisher's note:** Springer Nature remains neutral with regard to jurisdictional claims in published maps and institutional affiliations.

## Supplementary Material

Supplementary Information

Supplementary Table s3

Supplementary Table s4

Supplementary Table s5

Supplementary Table s6

## Figures and Tables

**Table 1:** List of significantly enriched cell differentiation-related GO terms (*q* < 0.05) identified in DEGs between adipogenic and myogenic cells.

Genes	U	D	Go terms	q value
Genes relating to cell differentiation and fate determination				
FGD4, LRFN2, JAG2, CEBPA, COL8A2, COL2A1, FSCB, TRIM55, ILDR1, NFATC2, PDGFA, SLIT2, TMPRSS11F, ARHGAP24, AGTR1, MLCK, COL8A1, MEGF10, MEOX2, PPFIA4, GPR39, ITGB6, STK17B, ECEL1, HES6, SLIT3, SCN4A, DPYSL2, RRAD, TNXB, NOTCH3, SRPK3, IGF2, FSCN2, CYR61, PKNOX2, TBX2, TRIM63, SEMA6B, OBSL1, LZTS2, LZTS1	36	6	cell differentiation (GO: 0030154);	0.031
JAG2, FZD6, HEY1, SIX2, PITX2, FZD4, HES6, HEYL	7	1	negative regulation of cell differentiation (GO: 0045596);	0.037
MYF5, SOX11, KLF14, ANKRD1, MYOG, HEYL, MEF2C, MET, WNT5B, CEBPA, PITX2, MAMSTR, SIX1, MYLPF, SMYD1	14	1	skeletal muscle cell differentiation (GO: 0035914);	0.005
			myotube differentiation (GO: 0014902);	0.013
			positive regulation of myotube differentiation (GO: 0010831);	0.037
			muscle cell fate specification (GO: 0042694);	0.029
			muscle cell fate determination (GO: 0007521);	0.021
			ureter smooth muscle cell differentiation (GO: 0072193);	0.041
SIX2, MYL2, MEF2C, TBX2, MEDAG, PITX3, PPARG, ARL4A	3	0	positive regulation of fat cell differentiation (GO: 0045600);	0.048
			brown fat cell differentiation (GO: 0050873);	0.050
NOTCH3, COL1A1, TRIM54, RHOB, SOX11, MEF2C, HEYL, NBL1, DMD, SIX1, HEY1, SIX2, HES6, TIMP2	15	0	positive regulation of neuron differentiation (GO: 0045666);	0.029
			regulation of neuron differentiation (GO: 0045664);	0.013
			neuron fate commitment (GO: 0048663);	0.050
Cell components of extracellular matrix, focal adhension, cytoskeleton				
SHROOM3, SGCG, KLHL40, RAPSN, CNN1, ABLIM3, STK17B, SGCA, KSR1, MICAL2, CSRP1, SRPK3, DMD, LOC102159034, DMPK, CAP2, MYZAP, RASA1, CORO2A, MUSK, PDGFA, FSCN1, FZD4, XIRP2, MET, FSCN2, TUBB2B, TPM1, ACTC1, ANK3, ACTA2, TUBA4A, TUBA1D, DES, TUBB6, ITGA2B, ITGB6, ITGA9, RND2, PDLIM3, SAMD14, STK26, MYO18A, MYH11, TPPP3, FGF13, FGF11, MLCK, PTPRK, SLC9A3R, TRIM54, LOC100626707, ACTN2, DPYSL2	50	5	cytoskeleton (GO: 0005856);	0.017
			actin cytoskeleton organization (GO: 0030036);	0.021
			integrin-mediated signaling pathway (GO: 0007229);	0.036
			actin filament bundle assembly (GO: 0051017);	0.029
			actin filament organization (GO: 0007015);	0.045
			microtubule polymerization (GO: 0046785);	0.021
			skeletal muscle thin filament assembly (GO: 0030240);	0.029
			actin filament depolymerization (GO: 0030042);	0.037
			actomyosin structure organization (GO: 0031032);	0.037
			actin-myosin filament sliding (GO: 0033275);	0.041
SCUBE1, JAG2, MMP15, LOC100155429, OLFML2B, BCAR3, FLRT3, FGFR2, TENM1, MEGF10, SCUBE2, FZD4, ADAMTS8, TENM3, ITGB6, SNED1, FAM83G, NOTCH3, COL1A1, FN1, TNC, SOD3	14	8	extracellular matrix (GO: 0031012);	0.003
PTPRK, LOC100620787, ACTN2	2	1	focal adhesion assembly (GO: 0048041);	0.045
Binding and cell adhension				
LRFN2, JAG2, LOC100155429, RASA1, COL2A1, COL15A1, FSCB, BCAR3, FLRT3, RHOB, FLT1, EFNB2, MEGF10, TGFBI, MCAM, NRCAM, C1QTNF3, AATK, NOTCH3, CYR61, LSAMP, COL11A2, LOC100620394, MUSK, ABLIM3, IL18, RND2, CYTIP, DMPK, PXM1, ITGA2B, NTM, PECAM1, ITGA9, TNXB, FN1, TNC, LOC100626707, LAMA2	30	10	cell adhesion (GO: 0007155)	5.48E-05
			regulation of cell adhesion (GO: 0030155)	0.046
MUSK, RHOB, ITGB6, EPDR1, TRIM54, COL8A2, FZD6, ABLIM3, RND2, SNED1, CDK3, TNXB, TNC, PPARG, PDGFRα	9	6	cell-matrix adhesion (GO: 0007160)	0.006
			cell-substrate adhesion (GO: 0031589)	0.014
CAP2, TPM1, NCALD, SHROOM3, MYOZ2, ANKRD1, XIRP1, MLCK, CNN1, ABLIM3, TAGLN, CXCR4, XIRP2, FLNC, MET, WIPF3, MYO1D, MICAL2, CSRP1, TNNT1, DMD, MYH11, NEXN, CORO2A, FSCN1, ACTN2, FSCN2, PKNOX2, ACTC1	29	0	actin binding (GO: 0003779)	1.25E-05
			actin filament binding (GO: 0051015)	0.008
			actin monomer binding (GO: 0003785)	0.028
			myosin binding (GO: 0017022)	0.034
			tropomyosin binding (GO: 0005523)	0.037
			integrin binding (GO: 0005178)	0.031
CELSR1, SAMD1, JAG2, CDH15, MMP15, RYR1, NCALD, TPD52, NINL, MYLPF, SLIT2, PCDH7, PCDH17, MYL2, PLA2G3, ACTN2, SCUBE2, CDH6, SLIT3, SGCA, EPDR1, NOTCH3, MLCK, CDH2, COL1A1, DMD, SCUBE1, C1R, SNED1, ATP2A3, RHBDL3	26	5	calcium ion binding (GO: 0005509)	0.008
RYR1, HRC, ACTN2, HOMER1, FGF13, FGF11	6	0	ion channel binding (GO: 0044325)	0.041
JAG2, LOC100155429, NOTCH3, LOC106506957, MAMDC2	5	0	glycosaminoglycan binding (GO: 0005539)	0.004
PDGFA, PDGFD, PDGFC, PDGFRα	3	1	platelet-derived growth factor receptor binding (GO: 0005161)	0.003
ALDH1A1, THRB	1	1	thyroid hormone binding (GO: 0070324)	0.003
Signaling pathways				
MYO1D, ADORA1, STK26, IL1RAP, FGF13	5	0	calcium-mediated signaling (GO: 0019722);	0.006
TNXB, NOTCH3, HEYL	2	1	Notch signaling pathway (GO: 0007219);	0.041
COL1A1, LRRC15	2	0	Wnt signaling pathway, calcium modulating pathway (GO: 0007223);	0.029
LRRC15, SBK1			positive regulation of Wnt signaling pathway, planar cell polarity pathway (GO: 2000096)	0.041
FGF11, COL1A1, PTGIR, PDGFRα	3	1	regulation of chemotaxis (GO: 0050920);	0.013
			negative regulation of chemokine-mediated signaling pathway (GO: 0070100);	0.041
WNT5B, EDN1, LRFN2, JAG2, RYR1, RASA1, FSCB, NCALD, SLIT2, EFNB2, ADAMTS14, RIPK4, GPC3, MEGF10, ABLIM3, SYTL2, SCUBE1, GPR68, ZNF423, AGTR1	16	4	cell surface receptor signaling pathway (GO: 0007166);	0.041
DMPK	1	0	negative regulation of platelet-derived growth factor receptor signaling(GO: 0010642)	0.041
Proteins modification				
MUSK, FLT1, LEPR, PDGFA, PDGFD, PDGFC, DDR1AATK, FGFR2, CNTFR, KSR1, PDGFRα	8	4	peptidyl-tyrosine phosphorylation (GO: 0018108);	0.021
			regulation of JUN kinase activity (GO: 0043506);	0.029
			peptidyl-tyrosin autophosphorylation (GO: 0038083);	0.029
			regulation of peptidyl-tyrosine phosphorylation (GO: 0050730);	0.032
TUBB2B, TUBA4A, TUBA1D, TUBB6	4	0	protein polymerization (GO: 0051258);	0.042
FSCB, MEGF10, SYTL2, HES6, C1QTNF3, AATK, ADORA1, IL1RAP, FGF13, FGF11, LRRC15, DMPK1, GPR68, TNXB	12	2	positive regulation of signal transduction (GO: 0009967);	0.026

U: number of up-regulated genes in myogenic cells, D: number of down-regulated genes in myogenic cells.
