# Supplementary material for: A potential regulatory network underlying distinct fate commitment of myogenic and adipogenic cells in skeletal muscle
Source: Sci Rep. 2017 Mar 9;7:44133. doi: 10.1038/srep44133 (PMC5343460; doi:10.1038/srep44133)
Supplement: Supplementary Information [file srep44133-s1.pdf]

Supplementary file

A potential regulatory network underlying distinct fate commitment of myogenic and adipogenic cells in skeletal muscle

Wenjuan Sun, Ting He, Chunfu Qin, Kai Qiu, Xin Zhang, Yanhong Luo, Defa, Li, Jingdong Yin*

State Key Lab of Animal Nutrition, College of Animal Science and Biotechnology, China Agricultural University, Beijing, 100193, China

* Correspondence: yinjd@cau.edu.cn

GN	Adi1	Adi2	Adi3	Myo1	Myo2	Myo3	FC
PAX7	0.065004	0.212667	0.139178	1.54331	0.728345	0.728345	10.76
Desmin	0.217444	0.205328	0.138593	1.333085	1.090018	0.576896	5.76
PDGFRα	1.168918	1.016378	0.814704	0.202573	0.169032	0.348473	4.54

Table s1. RT-qPCR analysis gene expression of myogenic markers (PAX7 and Desmin) and adipogenic marker (PDGFRα) and in myogenic and adipogenic cells. Fold change of PAX7 and Desmin were calculated by myogenic cells compared with adipogenic cells, Fold change of PDGFRα and Desmin were calculated by adipogenic cells compared with myogenic cells

Samples	Clean Reads	Uniq Mapped Reads	Mapped Ratio	Q 30(%)
Adi 1	14,161,374	10,825,575	84.12	95.54
Adi 2	12,292,339	9,509,932	85.08	95.76
Adi 3	13,242,191	10,133,368	84.29	95.55
Myo 1	12,676,074	9,693,087	84.01	95.50
Myo 2	11,091,882	8,536,897	84.40	95.24
Myo 3	12,297,105	9,544,873	85.39	95.70

Table s2. Summary of draft reads of adipogenic (Adi) and myogenic (Myo) cells from pig skeletal muscle by RNA-seq. The data of each type cells comes from three replicates.

GO Items	Ensemble ID	FDR	Log_2FC	Gene regulated	GN
Molecular Function：calcium ion binding (GO:0005509);	ENSSSCG00000000001	0.0010	2.18	up	CELSR1
	ENSSSCG00000000029	0.0426	-2.32	down	SCUBE1
	ENSSSCG00000000675	2.09E-23	-1.82	down	C1R
	ENSSSCG00000001699	0.0183	1.95	up	SAMD1
	ENSSSCG00000002642	0.0112	2.35	up	CDH15
	ENSSSCG00000002806	0.0003	2.78	up	MMP15
	ENSSSCG00000002961	0.0158	1.80	up	RYR1
	ENSSSCG00000006059	0.0004	1.80	up	NCALD
	ENSSSCG00000006156	2.93E-12	1.24	up	TPD52
	ENSSSCG00000007135	1.99E-06	1.31	up	NINL
	ENSSSCG00000007799	0.0001	1.45	up	MYLPF
	ENSSSCG00000008749	0.0001	1.32	up	SLIT2
	ENSSSCG00000008765	1.97E-05	1.08	up	PCDH7
	ENSSSCG00000009446	0.0015	1.71	up	PCDH17
	ENSSSCG00000009830	0.0487	2.30	up	MYL2
	ENSSSCG00000010023	0.0117	1.62	up	PLA2G3
	ENSSSCG00000010144	0.0001	1.33	up	ACTN2
	ENSSSCG00000014575	0.0159	1.36	up	SCUBE2
	ENSSSCG00000016157	1.98E-11	2.04	up	MYL1
	ENSSSCG00000016381	3.82E-09	-2.52	down	SNED1
	ENSSSCG00000016806	0.0138	2.64	up	CDH6
	ENSSSCG00000017012	0.0049	1.52	up	SLIT3
	ENSSSCG00000017583	0.0019	2.73	up	SGCA
	ENSSSCG00000017874	0.0246	-1.38	down	ATP2B
	ENSSSCG00000020963	0.0006	1.40	up	EPDR1
	ENSSSCG00000022850	0.0128	-1.39	down	RHBDL3
	ENSSSCG00000023899	0.0029	1.45	up	NOTCH3
	ENSSSCG00000024087	0.0003	1.74	up	MLCK
	ENSSSCG00000024790	0.0053	2.57	up	CDH2
	ENSSSCG00000025176	0.0028	1.40	up	COL1A1
	ENSSSCG00000028148	0.0080	1.42	up	DMD
Biological Process：calcium-mediated signaling (GO:0019722);	ENSSSCG00000001050	0.0407	2.21	up	EDN1
	ENSSSCG00000002961	0.0158	1.80	up	RYR1
	ENSSSCG00000006059	0.0004	1.80	up	NCALD
	ENSSSCG00000011695	8.74E-07	-1.50	down	AGTR1
	ENSSSCG00000015688	0.0001	3.75	up	CXCR4
	ENSSSCG00000017585	0.0238	-1.78	down	SAMD14

Table s7. List of DEGs which involved in calcium ion binding (GO: 0005509) and calcium-mediated signaling (GO: 0019722)

Gene symbol	Gen Bank accession no.	primer sequence	annealing temperature	Product size (bp)
CEBPA	XM_003127015	F: 5' GGCCAGCACACACACATTAGA3'	60	71
		R: 5'CCCCCAAAGAAGAGAACCAAG 3'		
DES	NM_001001535.1	F: 5‘AGCCGGATCAACCTCCCTAT3'	60	179
		R: 5’TGGCTTTAGAGCACCTCGTG3'		
DLK2	NM_001128466.1	F: 5' CACTTCCTCACCCTGTTCCAAT 3'	52	158
		R: 5' TACTCGTTCACACCGTCCCT 3'		
FZD6	NM_001315750.1	F: 5' CAATGTGAAGGATAAGAGCCGA 3'	60	166
		R: 5' GTGAACAAGCAGTGATGTGGAG 3'		
MEF2C	NM_001044540.1	F: 5' TATGGCAATCCCCGAAACTC 3'	60	146
		R: 5' GCCTGGTGGAATAAGAACT 3'		
Met	NM_001038008	F: 5' GTCTGCCTACAGTCTACAAG 3'	60	158
		R: 5' GGTCAAGGTACAGCTCTCAT 3'		
MYF5	NM_001278775	F: 5'AGTTCGGGGACGAGTTTGAG3'	60	232
		R: 5'-TCAAACGCCTGGTTGACCTT3'		
MYOG	NM_001012406.1	F: 5'CTGCTCACAGCTGACCCTAC3'	58.6	105
		R: 5'GGTTTCATCTGGGAAGGCCA3'		
PDGFD	XM_005667305.2	F: 5' TCCACTACAACATCGTCACA 3'	60	220
		R: 5' CTTCCGAAAACAAAAGCCT 3'		
PDGFRα	NM_001315756	F: 5' ATCGTGGAGAATCTGCTGCCTG 3'	63.4	216
		R: 5' GATGATGTAGCCGCTGTCTG 3'		
PPARG	NM_214379	F: 5' GAGGGCGATCTTGACAGGAA 3'	64	135
		R: 5' GCCACCTCTTTGCTCTGCTC 3'		
RHOB	NM_001123189	F: 5' GCTGATCGTGTTCAGTAAGG 3'	60	197
		R: 5' CCACCGAGAAGCACATAAGG 3'		
PAX7	XM_013992407	F: 5' CTCTGCCGCTACCAAGAGAC 3'	58	131
		R: 5' CTGAACATGCCTGGGTTCTC 3'		
GAPDH	NM_001206359	F: 5’TCGGAGTGAACGGATTTG3'	60	219
		R: 5’CCTGGAAGATGGTGATGG3'		

Table s8. List of the primer sequences used for RT-qPCR analysis.

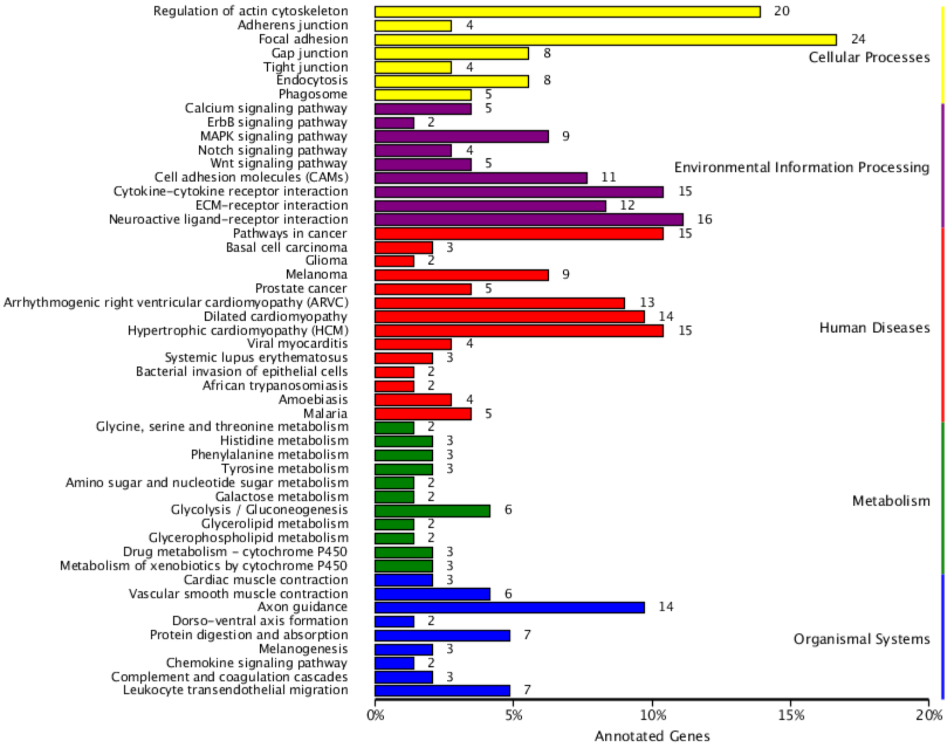

Figure s1. The top 50 pathways enriched with DEGs between adipogenic and myogenic cells. (Fifty pathways mainly clustered into 5 categories: cellular process, environment information processing, human disease, organismal systems and metabolism, the horizontal coordinate represented the percentage of annotated DEGs account for the total genes that included by correspond pathway) .

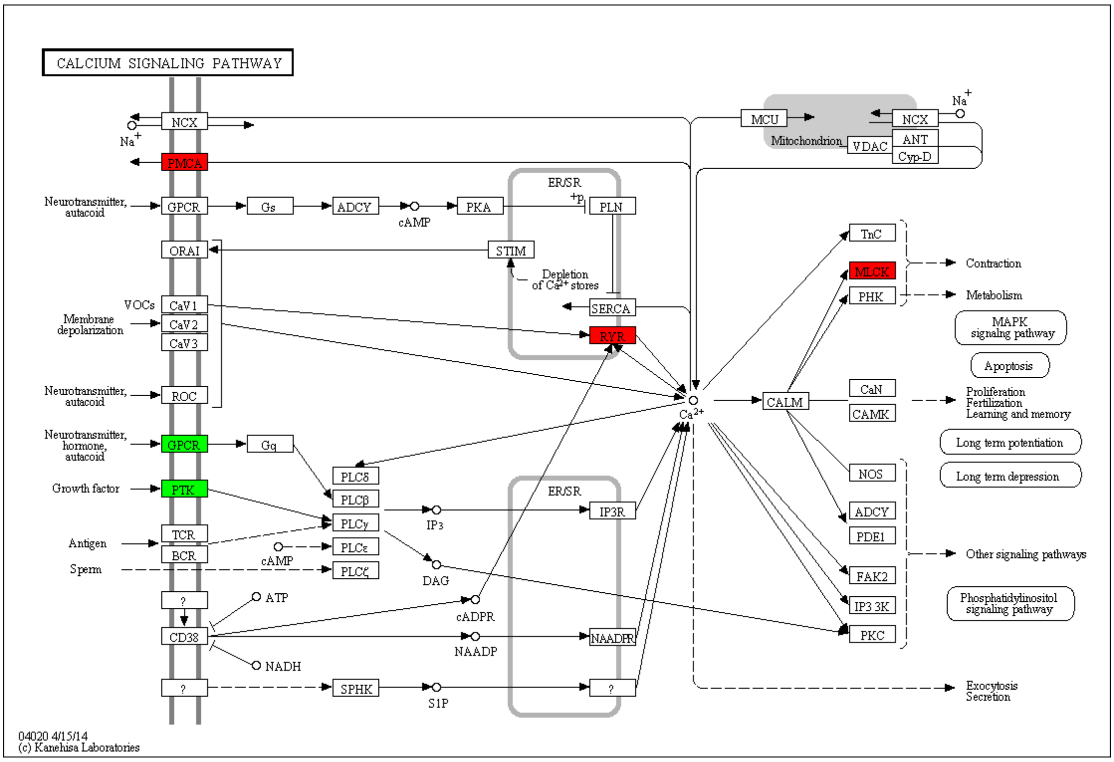

Figure s2. DEGs interaction in calcium signaling pathway. The colour of the nodes represent gene expression status (red: up regulated genes in myogenic cells, green: down regulated genes in myogenic cells, blue: gene set including both up and down regulated genes in myogenic cells) and the image was obtained by KEGG.

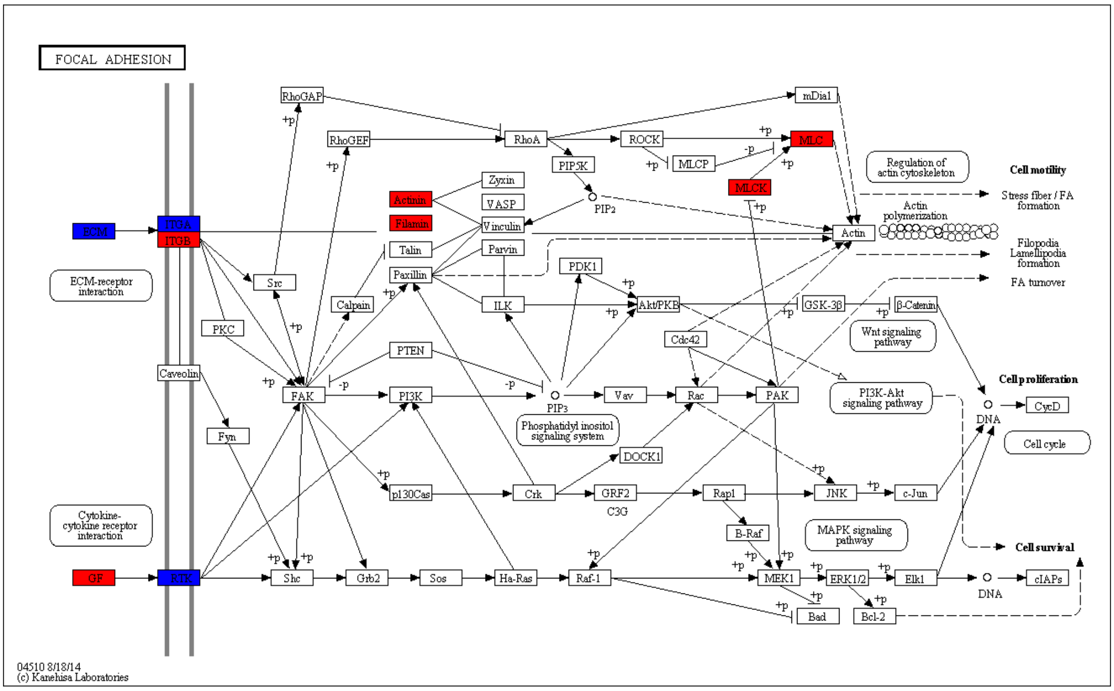

Figure s3. DEGs interaction in focal adhesion. The colour of the nodes represent gene expression status (red: up regulated genes in myogenic cells, green: down regulated genes in myogenic cells, blue: gene set including both up and down regulated genes in myogenic cells) and the image was obtained by KEGG.

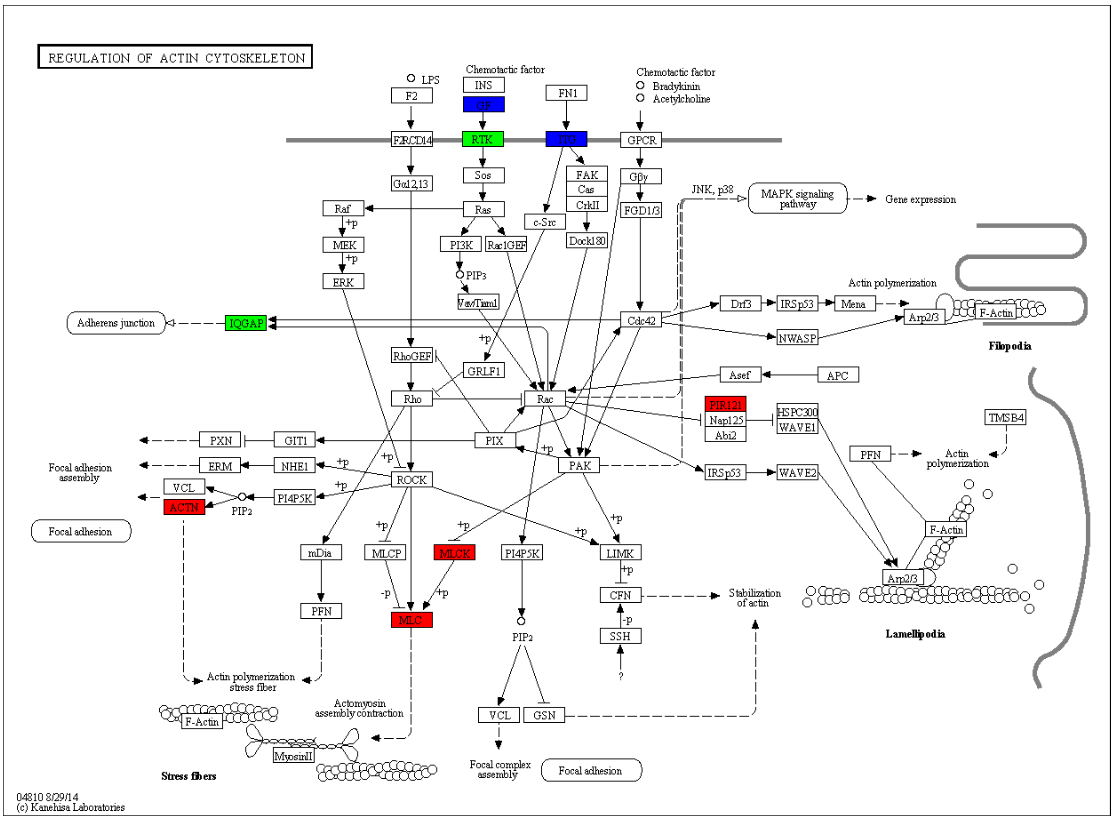

Figure s4. DEGs interaction in pathway of regulation of actin cytoskeleton. The colour of the nodes represent gene expression status (red: up regulated genes in myogenic cells, green: down regulated genes in myogenic cells, blue: gene set including both up and down regulated genes in myogenic cells) and the image was obtained by KEGG.

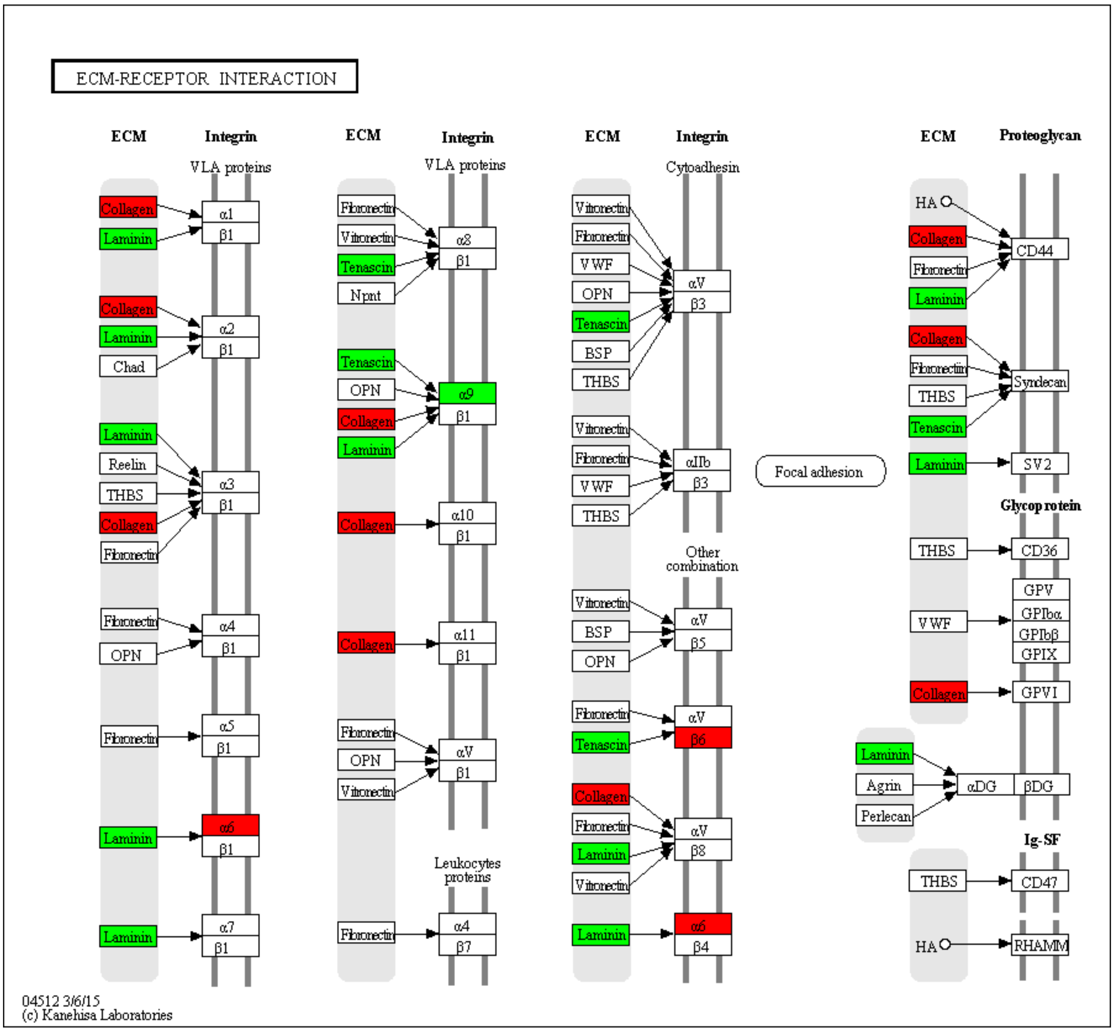

Figure s5. DEGs interaction in ECM-receptor interaction. The colour of the nodes represent gene expression status (red: up regulated genes in myogenic cells, green: down regulated genes in myogenic cells, blue: gene set including both up and down regulated genes in myogenic cells) and the image was obtained by KEGG.
